# The Effect of Citrus Essential Oils and Their Constituents on Growth of *Xanthomonas citri* subsp. *citri*

**DOI:** 10.3390/molecules22040591

**Published:** 2017-04-14

**Authors:** Hossein Mirzaei-Najafgholi, Saeed Tarighi, Morteza Golmohammadi, Parissa Taheri

**Affiliations:** 1 Department of Plant Protection, Faculty of Agriculture, Ferdowsi University of Mashhad, 9177948974 Mashhad, Iran; mirzaeih89@gmail.com (H.M.-N.); p-taheri@um.ac.ir (P.T.); 2 Iran Citrus Research Institute, 33546915 Ramsar, Iran; mgolm2009@gmail.com

**Keywords:** *Citrus aurantifolia*, *C. aurantium*, citral, synergistic effect, transmission electron microscopy

## Abstract

Citrus bacterial canker (CBC) caused by *Xanthomonas citri* subsp. *citri* (*Xcc*), is the most devastating of the citrus diseases worldwide. During our study, we found that Essential oils (EOs) of some citrus cultivars are effective on *Xcc*. Therefore, it prompted us to determine the plant metabolites responsible for the antibacterial properties. We obtained EOs from some locally cultivated citrus by using a Clevenger apparatus and their major constituents were identified by gas chromatography/mass spectrometry (GC-MS). The effect of *Citrus aurantium*, *C. aurantifolia*, *Fortunella* sp. EOs and their major constituents were evaluated against *Xcc*-KVXCC1 using a disk diffusion assay. Minimal inhibitory and bactericidal concentration of the EOs and their constituents were determined using the broth microdilution method. *C. aurantium*, *C. aurantifolia* Eos, and their major constituents including citral, linalool, citronellal, geraniol, α-terpineol, and linalyl acetate indicated antibacterial effects against *Xcc*. The *C. aurantifolia* EO and *citral* showed the highest antibacterial activity among the tested EOs and constituents with inhibition zones of 15 ± 0.33 mm and 16.67 ± 0.88 mm, respectively. Synergistic effects of the constituents were observed between α-terpineol-citral, citral-citronellal, citral-geraniol, and citronellal-geraniol by using a microdilution checkerboard assay. Transmission electron microscopy revealed that exposure of *Xcc* cells to citral caused cell wall damage and altered cytoplasmic density. We introduced *C. aurantifolia* and *C. aurantium* EOs, and their constituents citral, α-terpineol, citronellal, geraniol, and linalool as possible control agents for CBC.

## 1. Introduction

Citrus bacterial canker (CBC) is one of the most devastating citrus diseases which limits the cultivation of citrus plants in some tropical areas. The disease is caused by *Xanthomonas citri* subsp. *citri* (*Xcc*). The disease symptoms appear as crater-like lesions with a raised margin and sunken center surrounded by a yellow halo [1,2,3]. In severe infections, defoliation, twig dieback, badly blemished fruit, premature fruit drop, and reduction in quantity and quality of the fruits have been observed [4,5,6]. Different factors such as warmth, humidity, rainfall, wind, and leaf miners could affect the development and distribution of the disease. Several strategies including sanitation, resistant varieties, windbreaks, along with garden and plant treatment with bactericides have been proposed in order to control CBC [1,7,8]. Today, the use of bactericides are the most effective solution to manage CBC [8]. In order to increase public awareness regarding environmental problems associated with pesticides and antibiotics, a search for natural compounds to manage plant diseases seems inevitable [9,10]. Biological control by EOs could be an appropriate alternative for pesticides. These natural substances are aromatic, rarely colored, lipid soluble, and also soluble in organic solvents. They have been identified as safe constituents for prevention and therapy of plant-pathogenic microorganisms [9,11,12]. Antimicrobial properties of EOs are a result of the pivotal role of ketones, phenols and terpenes [13,14,15]. The antibacterial effect of EOs is associated with disturbance in coordinated ion flow of the cytoplasmic membrane via enhancement of membrane permeability or lipid depolymerisation. They also transform the structural makeup of the fatty acids, polysaccharides, proteins, and phospholipid layers in the membrane and cell wall of mitochondria. Some vital processes of the cells, such as energy conversion processes, nutrient processing, synthesis of macromolecules, and secretion of many growth regulators were impaired by EOs [13,14,16]. Fisher and Phillips have shown morphological changes of the blend effect of *C. sinensis* and *C. bergamia* against *Enterococcus faecium* and *E. faecalis* with the use of transmission electron microscopy (TEM) [17]. The citrus’s EO including lemon, bitter orange and kumkuat are widely used in medicine, cosmetic products, agriculture, and food industries [18]. Asnaashari et al. [19] introduced d-limonene (28.27%), α-terpineol (19.61%), *p*-cymene (8.58%), β-pinene (5.70%), 4-terpineol (4.76%), and linalool (2.39%) as major constituents of *C. aurantifolia* EO. In a survey conducted by Monsef-Esfahani et al. [20], the major constituents of *C. aurantium* were identified as geraniol (26.6%), α-terpineol (20.7%), linalool (15.4%) and benzene acetaldehyde. Several studies have reported the antibacterial effect of citrus EOs such as *C. aurantium* *C. sinensis*, *C. limon*, *C. reticulata*, and *C. aurantifolia* [21,22,23,24]. The geographical region can affect the EO composition and their antibacterial properties. Therefore, the purpose of this study was to explore the potential role of locally cultivated citrus EOs and their major constituents on growth of *Xcc*. Furthermore, we evaluated the role of citral as the most effective antibacterial constituent by TEM.

## 2. Results 

### 2.1. Antibacterial Activity Assays

**Figure 1:**
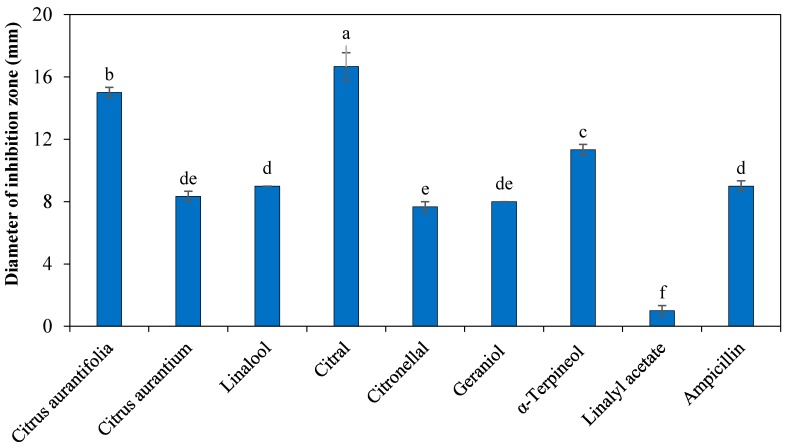
Antibacterial effect of essential oils and their constituents against *Xcc*-KVXCC1 isolates of *Xanthomonas citri* subsp. *citri*. Different letters indicate significant differences according to Duncan analysis using SPSS software (*p* = 0.05). All data represent means ± standard error of the mean (SEM) from 3 independent experiments. All data represent means ± standard error of the mean (SEM) from 3 independent experiments.

The effect of *C. aurantium*, *C. aurantifolia*, *Fortunella* sp. EOs, and their major constituents were investigated against *Xcc*-KVXCC1 using a disk diffusion assay. A broad variation in the antibacterial properties of EO and their constituents against Xcc-KVXCC1 were observed. Our results indicated that *C. aurantium*, *C. aurantifolia* EOs, and their major constituents including citral, linalool, citronellal, geraniol, α-terpineol, and linalyl acetate have antibacterial effects against *Xcc*-KVXCC1 (Figure 1). Antibacterial activity was not observed for *Fortunella* sp., limonene, geranyl acetate and *trans*-caryophllene. The *C. aurantifolia* and citral showed the highest inhibition zones of 15 ± 0.33 mm and 16.67 ± 0.88 mm, respectively. Furthermore, citral, α-terpineol and *C. aurantifolia* EO showed more antibacterial activity than ampicillin. On the other hand, linalyl acetate (1 ± 0.33 mm) demonstrated the weakest inhibitory effects against *Xcc*-KVXCC1.

### 2.2. GC-MS Analysis

EOs of *C. aurantium*, *C. aurantifolia*, and *Fortunella* sp. were obtained from leaves via a hydrodistillation method. The three EOs were analyzed by gas a chromatography/mass spectrometry (GC-MS) system and their chemical compositions were identified based on the comparison of the substance’s mass spectrum, with NIST mass spectra library stored in the GC–MS database and Adams literature (Table 1).

A total of 23, 16 and 28 EO constituents of *C. aurantifolia*, *C. aurantium* and *Fortunella* sp. were identified, representing 96.5%, 95.6%, and 96.8% of them, respectively. The main chemical constituents of the *C. aurantifolia* EO were limonene (47.2%), geraniol (9.8%), geranyl acetate (9.3%), linalool (6.7%), citral (5.2%), citronellal (4.9%), and *trans*-caryophyllene (3.9%). According to the result presented in Table 1, linalyl acetate (43.7%), linalool (25.9%), and α-terpineol (9.6%) were the prime constituents of *C. aurantium*. Germacrene D (17.4%), germacrene B (8.5%), elemol (7.9%), valencene (7.3%), *trans*-caryophyllene (7%), α-eudesmol (6.7%), and β-gurjunene (6%) were found to be the major constituents of *Fortunella* sp.

### 2.3. Determination of MIC and MBC

Minimum inhibitory concentration (MIC) and minimum bactericidal concentration (MBC) values of EOs and their constituents with bactericidal properties were determined and shown in Table 2. Different values of MIC for treatments against *Xcc*-KVXCC1 were observed. The MIC value for the EOs and their constituents ranged between 0.5 and 8.5. The lowest MIC value was related to the EO of *C. aurantifolia* and citral with 0.5 mg/mL and 0.375 mg/mL, respectively. In addition, the lowest and highest obtained MBC values for citral and linalyl acetate were 0.725 mg/mL and 14.5 mg/mL.

### 2.4. Synergist Assay

To investigate in vitro synergistic interaction of combinations of α-terpineol, citral, citronellal, geraniol, and linalool a microdilution checkerboard method was used. According to the obtained results, the synergism effect between α-terpineol-citral, citral-citronellal, citral-geraniol, and citronellal-geraniol were observed, and no antagonistic effect was showed between the tested constituents. In our study, the highest level of synergistic effect was related to a combination between citral-geraniol with 0/313 FIC index (Table 3).

### 2.5. Ultrastructural Changes of KVXCC1

**Figure 2:**
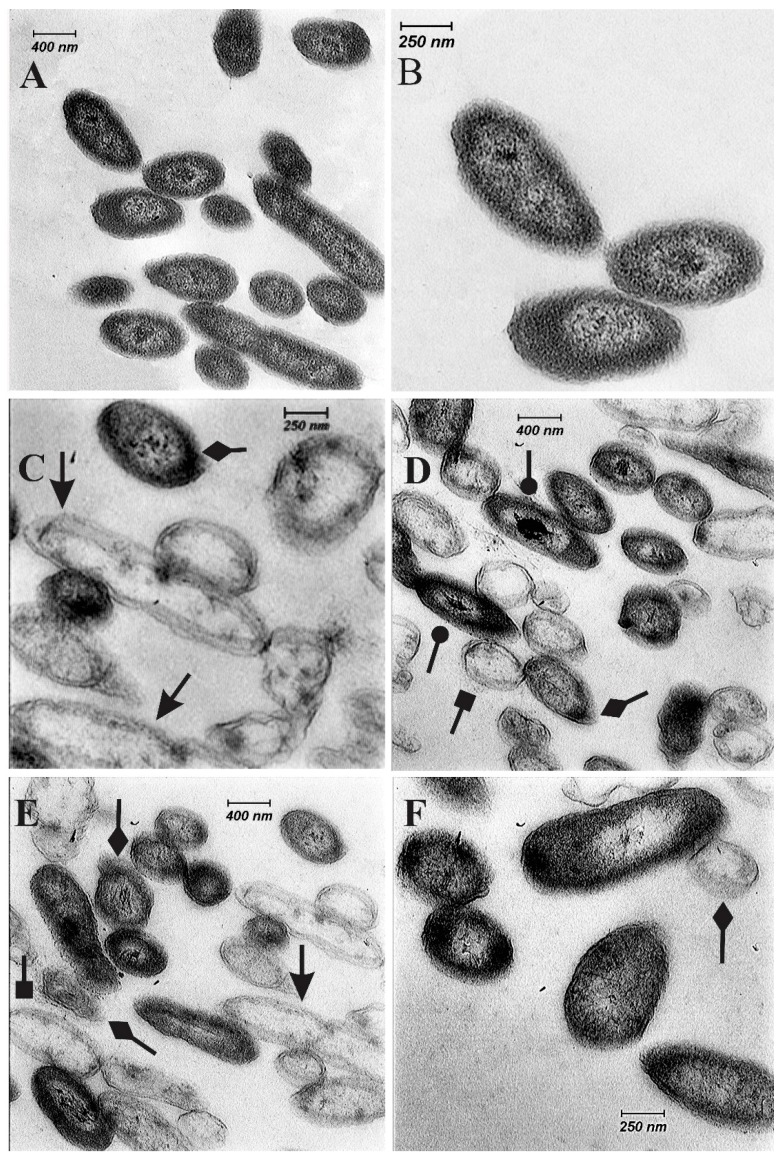
Transmission electron micrographs of *Xcc*-KVXCC1 isolate of *Xanthomonas citri* subsp. *citri* exposed to MIC value of citral for 4 h. (**A**,**B**), control with complete cell wall; (**C**) bacterial cells exposed to streptomycin sulfate (25 mg·mL^−1^); (**D**–**F**), bacterial cells exposed to citral with apparent the complete destruction of the cell (arrows), damage of cell wall (arrows with lozenge), alteration in cytoplasm density (arrows with square), swelling and colored nuclear area (arrows with circle).

Untreated cells of *Xcc*-KVXCC1 in a TEM study did not show any changes in the cell structure. The cells indicated obvious unified cell structure including a plasma membrane, cell wall, nuclear substances, and uniform cytoplasm (Figure 2A,B). While transformations such as complete destruction of the cell, cell wall damage, colored nuclear area, alteration in cytoplasm density, and swelling in cells treated with citral constituent were observed (Figure 2D–F).

### 2.6. Time-Kill Assay

**Figure 3:**
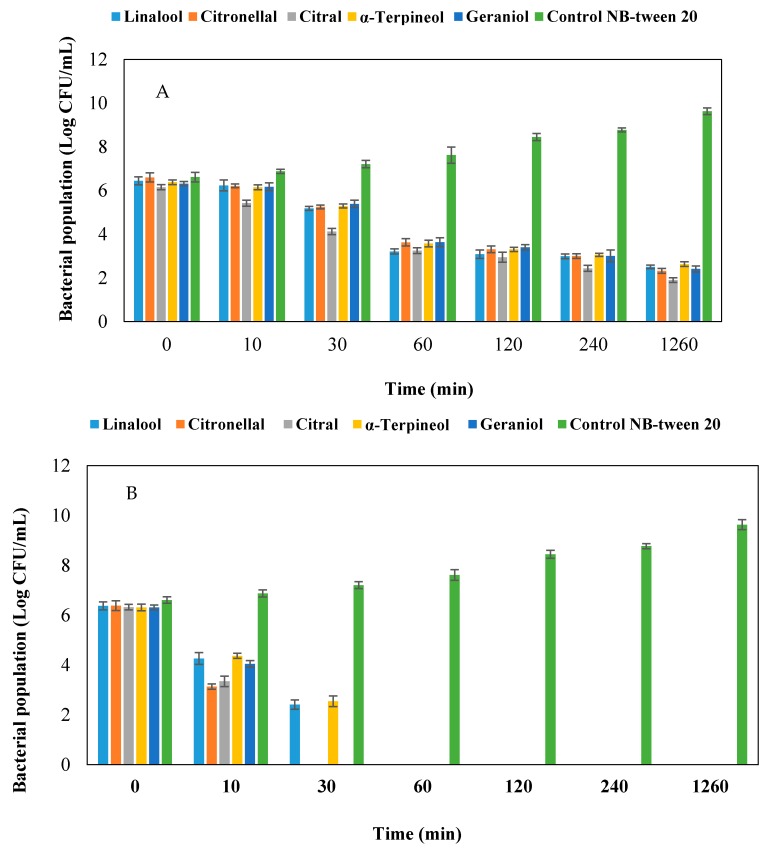
Time-kill kinetics of EO constituents against *Xcc*-KVXCC1 *Xanthomonas citri* subsp. *citri* at (**A**). MIC value and (**B**). 2.5× MIC value. All data represent means ± standard error of the mean (SEM) from three independent experiments.

To analyze the killing rate of EO constituents, time-kill assays were used at values of MIC, 2.5× MIC and 4× MIC. The killing curves of EO constituents are presented in Figure 3. Linalool and α-terpineol at 2.5× MIC value killed the bacterial cell during the first hour. While others constituents led to cell death after 0.5 h of incubation. All the constituents murdered bacterial cells after 10 min at 4× MIC concentration.

## 3. Discussion

CBC is one of the most destructive citrus diseases in citrus-producing areas. However, the disease management strategies including sanitation; resistant varieties, windbreaks, antibiotics, copper bactericides, and leafminer control could not completely eliminate the disease. The limitation in using bactericides resources and probability of appearance resistance to the bactericides (copper) prompted us to search for new bactericides with no side-effect on human health. For this purpose, we selected some citrus cultivars which were resistant to CBC. The EOs were obtained from the leaves of these cultivars and their constituents were identified using GC-MS analysis [9,11,12,25].

According to our results obtained by the disk diffusion assay, the EOs and constituents of *C. aurantifolia*, *C. aurantium*, citral, α-terpineol, geraniol, linalool, citronellal, and linalyl acetate indicated antibacterial activity. Chudasama and Thaker [9] showed that the *Cinnamomum cassia* EO has the highest antibacterial activity against *Xcc* with growth inhibition zones of 59 mm, while *C. aurantium* inhibited the growth of *Xcc* to a lesser amount of 8 ± 1 mm. In another study, Taiwo et al. [26] demonstrated antibacterial effect of *C. aurantifolia* and *Tithonia diversifolia* on clinical bacteria. They showed that the *C. aurantifolia* EO inhibited the growth of *Staphylococcus* sp., *Escherichia coli*, *Klebsiella* sp., *Proteus* sp., and *Pseudomonas* sp. [26].

Several researchers have described the antibacterial activity of citral, linalool, α-terpineol, citronellal, geraniol, and linalyl acetate against different pathogens [25,27,28,29,30,31]. The antibacterial effect of citral against *E. coli* K12, *Listeria innocua*, and *L. monocytogenes* have been reported by Belda-Galbis et al. [32] and Silva-Angulo et al. [33]. However, there are few reports about the antibacterial effects of EOs constituents against phytopathogenic bacteria, especially *Xcc*. [29]. The mode of action of citral against bacteria and fungi has been attributed to its reaction with the DNA, outer membrane, plasma membrane, and cell wall damage [28,29,34,35]. In the current study, we have shown the effect of citral on the plasma membrane, cell wall, and the nuclear substances (Figure 2).

The antibacterial effect of linalool was shown against Gram-positive and Gram-negative bacterial strains. Silva et al. [33] suggested, that linalool (terpene-alcohol) could cause disruption of the negatively charged Gram negative bacterial outer membrane. Geraniol has antibacterial activity against *E. coli* ATCC 25922, *P. mirabilis* ATCC 12453, *K. pneumoniae* ATCC 700603, *P. aeruginosa* ATCC 27853, and *S. aureus* ATCC 29213 [36]. Li et al. [37] indicated antibacterial effect of α-terpineol on growth of *E. coli*. Geraniol and α-terpineol are monoterpene alcohols. It may be through interaction with the membrane of cells that geraniol permeates into the interior of the cell and causes disturbance in the function of the bacteria [36]. Li et al. [37], by using of TEM, showed that during different time points, α-terpineol caused various change in the cell structure of *E. coli* such as plasmolysis, irregular cell shape, cell size decrease, cytoplasm lost, unequal division, and vacuolization of cells. Antibacterial activity of linalyl acetate attributed to a perturbation of the lipid fractions of bacterial plasma-membranes and the changes in membrane permeability [13].

We did not observe antibacterial activity of the geranyl acetate, limonene, and *trans*-caryophllene against *Xcc*-KVXCC. Limonene is in the terpene group. Based on the report of Nazzaro et al. [38], the terpene group had low or no antibacterial activity. For example, limonene had no antibacterial effect on *Pseudomonas mirabilis* and *P. aeruginosa*, while it had antibacterial effect on several pathogens [25,39,40].

By using GC-MS analysis, we identified 23, 16 and 28 molecules as major constituents of *C. aurantifolia*, *C. aurantium,* and *Fortunella* sp., respectively. Limonene (47.2%) constituted the highest percentage of *C. aurantifolia* EO. This result was in good agreement with other studies [22,39,40]. Similar to Babazadeh Darjazi [41] and Sarrou et al. [42], our data indicated that linalyl acetate (43.7%), linalool (25.9%), and α-terpineol (9.6%) were the major constituents of the *C. aurantium* EO, while these results were in contrast with the findings of Ellouze et al. [23], Caccioni et al. [43] and Rahimi et al. [44]. The EO obtained from the leaves of *Fortunella* sp. were found to contain 28 constituents with germacrene D (17.4%), germacrene B (8.5%), elemol (7.9%), valencene (7.3%), *trans*-caryophyllene (6.9%), α-eudesmol (6.7%), and β-gurjunene (6%) as the main constituents. The data obtained was broadly consistent with several studies [45,46]. The observed difference between the compound percentages and type of each EO’s compound (the type, number and percentage of constituents) depends on plant tissues such as leaves, peel and flower, developmental stage, extraction methods, temperature variation, different genotype, climatic conditions, season’s variation, relative humidity, and soil composition [13,23,42,44].

In the present study, the MICs for the selected EOs and their constituents were between 0.5 mg/mL and 8.5 mg/mL. Frassinetti et al. [24] evaluated the antimicrobial activity of EOs from *Citrus* spp. against ten strains of gram positive and negative bacteria. They showed that the MIC of the EOs against bacterial strains ranged between 15 µg/mL and 250 µg/mL and the lowest amount of MIC belonged to *C. lemon* against *Xcc*. In the study of Shi et al. [47], the MIC value of citral against *Cronobacter sakazakii* strains that was determined ranged from 0.27 mg/mL to 0.54 mg/mL. The citral in comparison with most EOs constituents is known to be a stronger antibacterial constituent. Park et al. [48] reported the MIC and MBC values of linalool and α-terpineol against periodontopathogens between 0.1 to 1.6 mg/mL. Furthermore, MIC of BIOLL+, a commercial extract obtained from citrus fruits, has a range from 10 ppm to 80 ppm for strains of *Brachyspira hyodysenteriae*, *Salmonella enterica*, and *E. coli* [16]. Li et al. [49] reported a MIC value of amicarthiazol against three amicarthiazol-resistant mutants and wild-type of *Xcc* between 400 µg/mL to 100 µg/mL.

**Figure 4:**
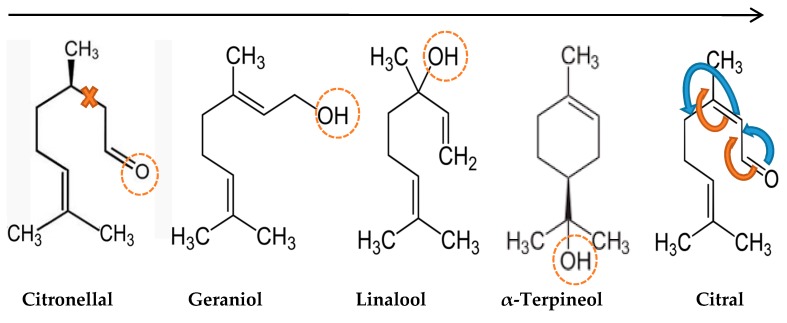
Schematic overview of the chemical structure of major essential oils. The straight black arrow shows the toxicity of the constituents subsequently. The curved red and blue arrows indicates different positions for double bands which makes two different isomers for citral. The circle dashed lines demonstrate the hydroxyl groups which probably attach to the bacteria. The cross red lines shows no possibility for citronellal to make any isomer.

In the present study, the synergistic effects among α-terpineol-citral, citral-citronellal, citral-geraniol, and citronellal-geraniol were observed (Figure 4). The combination of citral with α-terpineol, citronellal, and geraniol induced a synergistic activity against *Xcc*-KVXCC1 and in combination with linalool caused an additive effect. Many researchers have reported a synergistic relationship between citral-ε-PL, piperacillin-cinnamon bark oil, savory oil-chloramphenicol, savory oil-tetracycline, geraniol-chloramphenicol, and piperacillin-lavenderfor *E. coli* strains [36,47,50]. Using a combination of EO constituents could lead to a reduction in the effective dose of constituents and expand the antibacterial spectrum [51,52].

In a time killing assay, reduction in bacterial population size was different. The highest population reduction rate at MIC was in the range of log 6.15 to log 1.9 of citral constituent. While, the lowest population reduction rate was attributed α-terpineol. In this study, the highest population reduction size occurred at zero to one hour, and the killing effect of constituents had a descending trend. By increasing the concentration of the constituents, the time required for killing of the bacteria has decreased.

## 4. Materials and Methods

### 4.1. Bacterial Strains, Culture Conditions, and Chemical Constituents

The antibacterial effects of citrus EOs were screened on a hypervirulent strain of *Xcc* obtained from Kerman province, Iran (*Xcc*-KVXCC1). The bacteria was grown on NA (Nutrient Agar) at 28 °C for 24–48 h, unless stated otherwise. The bacterial cell concentration was synchronized to an optical density (OD) of 0.45 (1 × 10^8^ CFU/mL) at 600 nm in Nutrient Broth (NB). The EO constituents including geranyl acetate, linalyl acetate, limonene, *trans*-caryophllene, linalool, citronellal, citral, α-terpineol, and geraniol were prepared from Sigma-Aldrich (Brussels, Belgium).

### 4.2. Essential Oils Extraction 

The leaf samples of *C. aurantifolia*, *Fortunella* sp. and *C. aurantium* were collected from the plants grown at Ramsar greenhouses during the fruiting stage in the summer of 2014. All samples were washed with distilled water and dried out for 20 days at room temperature. EOs were extracted from leaf samples with hydrodistillation methods [53]. Fifty grams of crushed leaf samples were mixed with 500 mL of deionized water in a round-bottomed flask. Then the EO’s extraction was performed by using a Clevenger apparatus for 3 h. The extracted EOs were dried over anhydrous sodium sulfate and stored in refrigerator at 4 °C.

### 4.3. Disk Diffusion Assay

This method was used for primary screening of antibacterial properties of three EOs (*C. aurantium*, *C. aurantifolia,* and *Fortunella* sp.) and their major constituents against *Xcc*-KVXCC1 isolate [54]. A hundred microliter of the bacterial cultures (1 × 10^8^ CFU/mL) in NB were inoculated for 24 h to the NA media to prepare homogenous culture plates. Then blank paper discs (6 mm) smeared with 10 mg of each of the three EOs and nine constituents. Subsequently, plates were incubated at 28 °C for 48 h and thereafter the diameters of bacterial growth inhibition zones around discs were measured. Ampicillin (100 mg/mL) and distilled water were used as positive and negative controls, respectively. The experiments were done in triplicate.

### 4.4. Determination of Minimum Inhibitory Concentration (MIC) and Minimum Bactericidal Concentration (MBC)

MIC is defined as the lowest concentration of an antimicrobial agent which prevents the growth of bacteria after 24 h. The MIC of *C. aurantium*, *C. aurantifolia*, and their major constituents including citral, linalool, citronellal, α-terpineol, and geraniol were determined using microdilution and macro-dilution methods [54,55,56]. The overnight culture of *Xcc*-KVXCC1 strain in NB medium was prepared and the bacterial populations were adjusted to an optical density (OD) of 0.4 at 600 nm including 10^8^ CFU/mL bacteria. The 70 μL aliquots of NB medium were poured into the wells of ninety-six well tissue culture plates. Then 70 μL of NB medium including each of the EOs, or their tested constituents, were added to the wells at concentrations ranging from 30,000 µg/mL to 14 µg/mL. Finally, 10 μL of the bacterial suspensions were added to each well and the plates were incubated at 28 °C in a rotary shaker at 120 rpm.

To determine MBC, 30 μL of each well were cultured on the surface of NA medium and incubated for 48 h at 28 °C. The complete absence of bacterial growth was considered as MBC. To discover the exact MIC and MBC concentrations, a serial dilution in 5 mL falcon tubes including NB medium was performed between MIC and MBC concentrations that were obtained by a micro-dilution method. The experiment was performed in triplicate.

### 4.5. GC-MS Analysis

GC analyses were performed using a gas chromatograph (Thermo Quest 2000, Lancashire, UK) equipped with a DB-5 fused silica column (30 m × 0.2 mm, film thickness 0.32 μm). The analyzed condition of EOs consisted of: injection temperature: 260 °C, interface heating: 300 °C, ion source heating: 200 °C, EL mode: 70 eV, scan range: 41–450 amu and scan time 0.50 s. The GC-MS oven temperature was 55–120 °C (3 °C/min), 120–200 °C (4 °C/min), 200–220 °C (6 °C/min) and 220 for 5 min [42]. Percentages were calculated by electronic integration of FID, peak areas without the use of a response factor correction. GC/MS analyses were carried out on a Saturn-3400 GC-MS system (Thermo Quest Finningan, Lancashire, UK) equipped with a DB-5 fused silica column (30 m × 0.2 mm, film thickness 0.32 μm); with similar temperature programmed as in GC, transfer line temperature 260 °C, carrier gas He with a linear velocity of 31.5 cm/s, split ratio 1/60, ionization energy 70 eV.

The constituents of the essential oils were identified by calculation of their retention indices under temperature-programmed conditions on a DB-5 column under the same chromatographic conditions. Identification of individual compounds was made by comparison of their mass spectra with those of the internal reference mass spectra library or with authentic compounds and confirmed by comparison of their retention indices with authentic compounds or with those reported in the literature [57]. For quantification purposes, relative area percentages obtained by FID were used without the use of correction factors.

### 4.6. Identification of Synergistic Effects between EOs Constituents

The microdilution checkerboard method on 96-well plates was used to evaluate synergism effects of EO constituents (α-terpinolene, linalool, citronellal, geraniol, and citral) [58]. Seventy microliter of each dilution (2× MIC, 1× MIC, 1/2, 1/4, 1/8, 1/16, 1/32, and 1/64 MIC) were dispensed to each row, and then 70 μL of another constituent added to each row of the wells in the direction perpendicular to the previous constituents in different dilutions. Finally, 10 μL of NB media containing 1 × 10^8^ CFU/mL was added to each well. The plates were incubated at 28 °C on a rotary shaker at 125 rpm for 24 h. All treatments were triplicated. A fractional inhibitory concentration index (FICI) of the dual combination of EO constituents were calculated by using the following formula:

$$\mathrm{FICI}=\mathrm{FIC}\;A\;+\;\mathrm{FIC}\;B\;=\;\frac{\mathrm{MIC}\;A\;\mathrm{combined}}{\mathrm{MIC}\;A\;\mathrm{alone}}\;+\;\frac{\mathrm{MIC}\;B\;\mathrm{combined}}{\mathrm{MIC}\;B\;\mathrm{alone}}.$$

Interaction of the combination of two substances was defined as a synergistic effect, if the FIC index was ≤0.5, additively if 0.5 < FICI < 1, indifferently if 1 < FICI ≤ 4, and antagonistically if FICI > 4.

### 4.7. Time-Kill Assay

A time-kill assay of EO constituents was carried out at 1× MIC, 2.5× MIC, and 4× MIC using the method of Gerits et al. [59]. The *Xcc*-KVXCC1 strain was inoculated to NB at 10^5^ CFU/mL and incubated in orbital shakerat 28 °C for 24 h. Subsequently, a 100 μL aliquot was removed from each sample at different time points (0, 10, 30, 60, 120, 240, and 1260 min) and serially diluted in MgSO_4_ (10 mM). The number of viable cells was determined by the plate count technique. The distilled water and inoculated NB without EO constituents was used as the control. The experiments were performed in triplicate.

### 4.8. Preparation of Cells for TEM

In order to prepare *Xcc*-KVXCC1 cells for evaluating the citral effect on bacteria, overnight cultures of *Xcc*-KVXCC1 at 28 °C in NB was prepared for transmission electron microscopy (Leo 912 AB) analyses [12]. The citral was added to the bacterial culture at the MIC value. The bacterial suspensions were centrifuged at 6000 rpm for 7 min and cell pellets were pre-fixed on glutaraldehyde solution 2.5% (with cacodylate buffer 0.1 mol/L) for 24 h at −4 °C. Then samples were washed three times for 10 min with cacodylate buffer and post-fixed on 1.0% osmium tetroxide. The samples, which were washed on cacodylate buffer for 15, 30, and 60 min, were dehydrated in a graded ethanol series (30, 50, 70, 80, and 100%). Later, samples were embedded in propylene oxide and resin in ratios of 1:3, 1:1, 3:1, and pure resin for 12, 4, 12 and 24 h, the sample blocks were polymerized in an oven at 60 °C, for 48 h. Then sections of 75 nm by ultramicrotomy (Ultracut R, Leica, Wetzlar, Germany) were prepared and stained with 1% uranyl acetate and lead (II) nitrate. In this study, no-treated bacterial cells or streptomycin were used as negative and positive control, respectively.

## 5. Conclusions

Working on antibacterial and synergistic combinations in order to increase efficiency and prevent the development of resistance in bacteria against bactericides will help to control the disease caused by pathogens. *C. aurantifolia* and *C. aurantium* showed stronger antibacterial activity against the Xcc-KVXCC1 strain. The main constituents of EOs including citral, linalool, α-terpineol, and geraniol have shown proper antibacterial effect. Many studies have investigated synergistic effects of antibiotics and EOs against different pathogens. So far, no studies have been done about synergistic effect of the constituents α-terpineol, citral, citronellal, geraniol, and linalool against *Xcc*. In our study, synergistic, additive, and indifferent effects were showed, but no antagonistic effects were observed among treatments. Based on the present study, we introduced *C. aurantifolia* and *C. aurantium* EOs and constituents citral, α-terpineol, citronellal, geraniol, and linalool as possible control agents for CBC.

## Figures and Tables

**Table 1:** Chemical composition of the essential oils of *Citrus aurantifolia*, *C. aurantium* and *Fortunella* sp.

No.	*Citrus aurantifolia*			*Citrus aurantium*			*Fortunella* sp.		
	RI	Constituent	Percent	RI	Constituent	Percent	RI	Constituent	Percent
1	935	α-pinene	0.3	973	sabinene	2.1	952	camphene	0.2
2	978	β-pinene	3.4	978	β-pinene	1.7	1100	linalool	0.6
3	1028	limonene	47.2	992	β-myrcene	2.3	1168	borneol	0.9
4	1039	*cis*-β-ocimene	0.5	1028	limonene	2.3	1188	α-terpineol	0.4
5	1100	linalool	6.7	1042	*cis*-β-ocimene	2.3	1259	*trans*-geraniol	0.1
6	1133	*cis*-limonene oxide	0.2	1086	α-terpinolene	0.2	1335	δ-elemene	9.4
7	1142	*trans*-limonene oxide	0.1	1100	linalool	25.9	1351	α-cubebene	0.6
8	1154	citronellal	4.9	1188	α-terpineol	9.6	1377	α-copaene	1
9	1201	(4*Z*)-decanal	0.1	1250	geraniol	1.7	1392	β-elemene	5
10	1227	nerol	0.1	1257	linalyl acetate	43.7	1417	*trans*-caryophyllene	7
11	1250	geraniol	9.8	1367	neryl acetate	0.8	1440	β-gurjunene	6
12	1341	citral	5.2	1386	geranyl acetate	2.5	1445	aromadendrene	0.3
13	1357	citronellyl acetate	0.3	1417	*trans*-caryophyllene	0.1	1448	α-humulene	2.5
14	1367	neryl acetate	2.1	1448	α-humulene	0.2	1481	germacrene d	17.4
15	1386	geranyl acetate	9.3	1508	α-farnesene	0.1	1487	β-selinene	1.6
16	1417	*trans*-caryophyllene	3.9	1725	farnesol	0.1	1492	δ-selinene	0.5
17	1419	*cis*-α-bergamotene	0.3				1490	valencene	7.3
18	1448	α-humulene	0.4				1507	(*E*)-α-farnesene	0.4
19	1458	(*E*)-β-farnesene	0.7				1509	ϒ-cadinene	2
20	1582	caryophyllene oxide	0.8				1519	δ-cadinene	1.9
21	1677	(*Z*)-nerolidyl acetate	0.1				1549	elemol	7.9
22	1725	(2*E*,6*Z*)-farnesol	0.1				1553	germacrene B	8.5
23							1563	(*E*)-nerolidol	1.5
24							1581	spathulenol	1.2
25							1590	globulol	2.1
26							1624	10-epi-γ-eudesmol	1.3
27							1649	β-eudesmol	2.5
28							1693	α-eudesmol	6.7
total			96.5			95.6			96.8

**Table 2:** Minimum inhibitory concentration (MIC) and minimum bactericidal concentration (MBC) value of EOs and their main constituents against *Xcc*-KVXCC1 isolates of *Xanthomonas citri* subsp. *citri*.

Essential Oil/Compound	MIC mg/mL	MBC mg/mL
*Citrus aurantifolia*	0.5 b	0.9 b
*Citrus aurantium*	1.3 g	2 g
α-terpineol	0.625 c	1.1 c
citral	0.375 a	0.725 a
citronellal	1 f	1.41 f
geraniol	0.9 e	1.325 e
linalool	0.85 d	1.225 d
linalyl acetate	8.5 h	14.5 h

Means with a different letter in a row are statistically significant at 1% level.

**Table 3:** FIC index EO of constituents against *Xcc*-KVXCC1 isolates of *Xanthomonas citri* subsp. *citri*.

Compound	FIC Index	Activity
α-terpineol-citral	0.44	Synergistic
α-terpineol-citronellal	1	Indifferent
α-terpineol-geraniol	0.625	Additive
α-terpineol-linalool	1	Indifferent
citral-citronellal	0.5	Synergistic
citral-geraniol	0.313	Synergistic
citral-linalool	0.625	Additive
citronellal-geraniol	0.625	Additive
citronellal-linalool	0.5	Synergistic
geraniol-linalool	0.625	Additive
